# Comparison of Pharmacokinetics and Tissue Distribution Characteristics of Three Diterpenoid Esters in Crude and Prepared Semen Euphorbiae

**DOI:** 10.1155/2021/7402120

**Published:** 2021-08-16

**Authors:** Hui-Nan Wang, Pei-Hua Wang, Zi-Ye Yang, Gui-Mei Zhang, Meng-Yu Chen, Ming-Rui Jiang, Zhu-Zhu Yue, Zhi-Cheng Wang, Jing-Qiu Zhang, Yan-Hua Cao, Hong An, Ying-Zi Wang

**Affiliations:** ^1^School of Chinese Materia Medica, Beijing University of Chinese Medicine, Beijing 102488, China; ^2^Department of Pharmacy, Shandong Provincial Hospital Affiliated To Shandong First Medical University, Jinan 250021, China

## Abstract

**Background:**

Semen Euphorbiae (SE) and Semen Euphorbiae Pulveratum (SEP) have a long history of medicinal use. SEP is the processed product of SE; both ancient and modern studies have shown that SEP has a lower toxicity compared to SE. To clarify the influence of processing on the pharmacological properties of SE and SEP, a study was carried out to compare the pharmacokinetics and distribution characteristics of three active compounds after oral administration of SE and SEP extracts.

**Methods:**

A UPLC-MS/MS method was established to simultaneously determine the contents of Euphorbia factors L_1_, L_2_, and L_3_ in rat plasma and mouse tissues after an oral administration of crude and processed SE with approximately the same dosage. Plasma and heart, liver, spleen, lung, kidney, and colon tissue samples were treated with ethyl acetate and separated by gradient elution on a C18 column with a mobile phase of 0.1% formic acid and methanol.

**Results:**

The established method had good selectivity, linear range, accuracy, precision, stability, matrix effect, and extraction recovery. The area under the concentration time curve, time to maximum concentration, maximum concentration, half-life of elimination, and mean retention time of plasma samples in SEP-treated group decreased, and the clearance in SEP-treated group increased. Moreover, the active component concentrations in colon, liver, and kidney tissues were more followed by those in the heart, lungs, and spleen.

**Conclusion:**

These results indicate that the processing could influence the pharmacokinetics and tissue distribution of Euphorbia factors L_1_, L_2_, and L_3_ after oral administration of crude and processed SE. The data obtained may lay a foundation for the clinical use of SE and for further study on the processing mechanism of SE.

## 1. Introduction

Semen Euphorbiae (SE) is the dried mature seed of *Euphorbia lathyris* L., which is a kind of traditional Chinese medicine (TCM) that is widely used in the treatment of hydroncus, ascites, anuria, astriction, menostasis, and scabies [1, 2]. Modern pharmacological studies have shown that SE has several pharmacological activities, including laxative effects, antitumor effects, reversal of tumor multidrug resistance effects, anti-inflammatory activity, and whitening effects [3–5]. However, its toxicity greatly limits its further development and clinical application. Semen Euphorbiae Pulveratum (SEP) is the processed product of SE, prepared by removing the oil from the SE; both ancient and modern studies have shown that SEP has a lower toxicity compared to SE [6, 7]. Considering that processing can change the content of the active ingredients and affect their pharmacokinetics [8, 9], it is necessary to study the pharmacokinetics differences of the active components of SE before and after processing.

Phytochemical investigation reveals that SE contains diterpenoids, sterols, coumarins, flavonoids, volatile oils, fatty oils, and many other chemical components [5]. Diterpene esters are the primary and active components of SE, among which Euphorbia factors L_1_, L_2_, and L_3_ are three most major active diterpene esters [10]. These three components are found in relatively high amounts and have significant pharmacological effects such as laxative, anti-inflammatory, and antitumor effects [11–13].

Pharmacokinetic studies are helpful to explain and predict the absorption, distribution, and excretion of active ingredients in *vivo* [14, 15]. HPLC has been used to study the pharmacokinetics of Euphorbia factor L_1_ [16], while LC-MS/MS method has been used to determine the pharmacokinetic properties of three diterpene esters after oral administration of the SE extract [17]. However, these methods exist with some limitations, containing low sensitivity, long running time, large sample size, and complex sample pretreatment [18]. Thus, it is necessary to establish a sensitive and quantitative method to simultaneously determine the differences in the concentration and distribution of Euphorbia factors L_1_, L_2_, and L_3_ in crude and prepared SE in rat plasma and mouse tissues.

Our study reports a rapid and sensitive UPLC-MS/MS method to simultaneously determine the contents of Euphorbia factors L_1_, L_2_, and L_3_ in rat plasma and heart, liver, spleen, lung, kidney, and intestinal tissues of mice. This method has been applied to determine the pharmacokinetics and tissue distribution characteristics of crude SE and its processed products in order to clarify the influence of processing on the pharmacological properties of SE and to lay a foundation for further study of SE.

## 2. Materials and Methods

### 2.1. Chemicals and Materials

SE was bought from Anhui Bozhou Huqiao TCM Herbal Pieces plant in Jiangxi Province, China (Lot No. 1203070692), and identified by Professor Chunshen Liu from the School of Chinese Materia Medica, Beijing University of Chinese Medicine. Petroleum ether extracts of SE and SEP were made in the laboratory. The reference standards of Euphorbia factor L_1_ (Lot No. 111789-200901), Euphorbia factor L_2_ (Lot No. 111790-200901), and Euphorbia factor L_3_ (Lot No. 111791-200901) have the purity of 99.3%, 98.5%, and 98.6%, respectively, which were obtained from China Institute of Pharmaceutical and Biological Products in Beijing, China, while wogonin (Lot No. YS0925SA13) with the purity of more than 98% was obtained from Shanghai Yuanye Biotechnology Company LTD. in Shanghai, China.

### 2.2. Animals

Healthy Sprague-Dawley (SD) rats weighing 250 ± 20 g and Kunming mice (KM) weighing 20 ± 2 g were bought from SPF Biotechnology Company Ltd., in Beijing, China (Lot No. 2016-0002). The animals have free access to water and food. The animal experiment was carried out strictly according to the Provisions on the Administration of Experimental Animals issued by the Ministry of Science and Technology of the People's Republic of China. The program was authorized by the Experimental Animal Ethics Committee of Beijing University of Chinese Medicine, and this study was carried out in strict compliance with the ARRIVE guidelines [19].

### 2.3. UPLC-MS/MS Conditions

The UPLC tandem quadrupole mass spectrometer (MS) (Acquity UPLC Xevo TQ-S, Waters, USA) and ACQUITY UPLC^®^ BEH C18 column (1.7 *μ*m, 50 mm × 2.1 mm) were used. A gradient elution procedure of 0.1% formic acid (a)-methanol (b) was used for analysis. The elution procedure was as follows: 0-1 minute (min) (40% b); 1-2 min (40–75% b); 2–4 min (75–85% b); 4–5 min (85–100% b); 5–7 min (100% b); 7–7.5 min (100–40% b); 7.5–10 min (40% b). The flow rate was 0.4 mL·min^−1^, the column temperature was 40°C, the detection wavelength was 275 nm, and the injection volume was 2 *μ*L. The sample extracts were maintained in the autosampler at 10°C.

**Figure 1:**
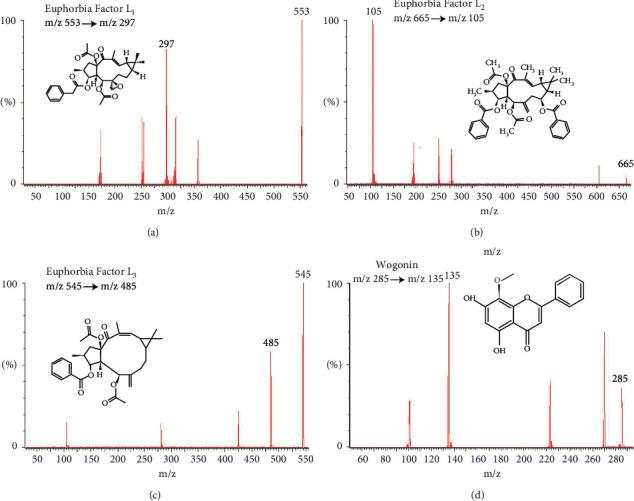
Full scan ion spectrum of Euphorbia factor L_1_ (a), Euphorbia factor L_2_ (b), Euphorbia factor L_3_ (c), and IS (d) in positive ion mode.

Electrospray ionization (ESI) source was used for MS detection under positive ion mode. The multi-ion monitoring (MRM) mode was used for scanning. The conditions of MS were as follows: desolvent gas flow rate, 800 L·hour^−1^ (L·h^−1^); desolvent gas temperature, 400°C; cone gas flow, 150 L·h^−1^; capillary voltage, 3.0 kV. Quantification was performed at m/z 553⟶297 (Euphorbia factor L_1_), m/z 665⟶105 (Euphorbia factor L_2_), m/z 545⟶485 (Euphorbia factor L_3_), and m/z 285⟶135 (wogonin, internal standard, IS). The MS diagram of these components is shown in Figure 1.

### 2.4. Preparation of Standard Solutions and Quality Control Samples

The reference standards, including those of Euphorbia factors L_1_, L_2_, and L_3_, were each weighed and diluted with methanol, yielding stock solutions with concentrations of 1.002, 1.010, and 1.002 mg·mL^−1^. The stock solutions of Euphorbia factors L_1_, L_2_, and L_3_ were then diluted with methanol to obtain the standard solutions with concentrations of 10.02, 10.10, and 10.02 *μ*g·mL^−1^, respectively.

The wogonin was precisely weighed and diluted to 25 mL using methanol, yielding a concentration of 2.016 *μ*g·mL^−1^ and then diluted with methanol to obtain the quality control (QC) solution with a concentration of 20.16 ng·mL^−1^.

### 2.5. Sample Preparation

SE (500 g) and SEP (500 g) were extracted using the method described by Zhu et al. [20, 21]. SE and SEP were weighed and reflux extracted for three times using 95% ethanol. The extracts from each reflux were filtered, the filtrates were combined and concentrated, followed by the addition of water to form a suspension. The suspension was extracted with an equal volume of petroleum ether for 3 times, and the supernatant was concentrated to obtain the SE extract with a concentration of 5.53 g·mL^−1^ for SE and the SEP extract with a concentration of 5.74 g·mL^−1^ for SEP. SE extract contains Euphorbia factor L_1_ of 4.80 mg·g^−1^, Euphorbia factor L_2_ of 2.21 mg·g^−1^, and Euphorbia factor L_3_ of 4.89 mg·g^−1^, and SEP extract contains Euphorbia factor L_1_ of 2.75 mg·g^−1^, Euphorbia factor L_2_ of 0.97 mg·g^−1^, and Euphorbia factor L_3_ of 2.30 mg·g^−1^, respectively.

### 2.6. Pretreatment of Plasma and Tissue Samples

Plasma (100 *μ*L) from each group was collected and 100 *μ*L wogonin solution with a concentration of 20.16 ng·mL^−1^ and 1 mL ethyl acetate were added. The samples were then whirled for 5 min, centrifuged at 8000 g for 10 min. Next, 800 *μ*L of supernatant fluid was taken out and the remaining liquid was vortexed for 5 min and centrifuged at 8000 g for 10 min. Then, all of the supernatant fluid were combined and dried under nitrogen at 37°C. Subsequently, the dry extract was dissolved in 100 *μ*L of methanol, whirled for 1 min, centrifuged at 8000 g for 5 min, and the supernatant fluid was analyzed using UPLC-MS/MS. The tissue samples were prepared similarly.

### 2.7. Method Validation

The method was totally validated in accordance with the US FDA guidelines [22].

#### 2.7.1. Selectivity

The selectivity was estimated by comparing chromatograms of blank plasma (tissue) samples without drugs, blank plasma without drugs added with IS solution, blank plasma (tissue) samples with IS solution and mixed standard solution, and plasma (tissue) samples from rat plasma or mouse tissues after the oral administration of SE and SEP, respectively.

#### 2.7.2. Linear Range and Limit of Quantitation (LOQ)

Standard curves were constructed with ten concentrations (1, 2, 5, 10, 25, 50, 100, 200, 400, and 800 ng·mL^−1^) of standard solution and blank plasma samples and then subjected to sample pretreatment. The linear range of each curve was estimated by means of the correlation coefficient (*r*). The LOQ was defined as the minimum quantity of the measured substance in the sample, which reflected whether the method used was sensitive and well suited for quantitative detection.

#### 2.7.3. Precision and Accuracy

The relative standard deviation (RSD) and relative error (RE) were used to estimate the precision and accuracy. Three QC samples (low, medium, and high) were used to test the intra- and interday accuracy and precision on the same day and three consecutive validation days.

#### 2.7.4. Stability

Stability of the three components in rat plasma and mouse tissues was evaluated in low, middle, and high concentrations of QC samples using three replicates. The conditions are as follows: room temperature for 30 min, three freeze-thaw cycles, and postpreparative samples stored in the automatic injector at 4°C for 24 h.

#### 2.7.5. Matrix Effect and Extraction Recovery

The extraction recoveries of the three components were defined by comparing the peak areas of the mixed standard solution and internal standard solution preadded to blank plasma with that postadded to blank plasma. The matrix effect was assessed by comparing the peak areas of the mixed standard solution and internal standard solution postadded to the blank plasma with those in the mobile phase.

### 2.8. Design of the Pharmacokinetic Study

A total of 12 male SD rats were divided into SE group and SEP group. The rats were fasted for 12 h before administration but could drink freely. SE and SEP extracts were dissolved in physiological saline to obtain a test solution containing 3.75 g of SE or SEP in 1 mL solution. Accordingly, six rats in each group were separately given SE and SEP test solution at a dose of 30 g·kg^−1^ by intragastric administration. 0.5 mL blood samples were taken from the fundus venous plexus at the specified time points (5 and 15 min, 0.5, 1, 1.5, 2, 4, 8, 12, 24, 36, and 48 h) before and after administration. At the end of the experiment, all rats were lightly anesthetized before euthanasia. The blood samples were immediately centrifuged at 8000 g for 10 min. The plasma was stored at −20°C until further analyses.

### 2.9. Design of the Tissue Distribution Study

SE and SEP extracts were dissolved in physiological saline to obtain a test solution containing 1.60 g of SE or SEP in 1 mL solution. A total of 72 male KM mice were orally administered SE and SEP test solution at a dose of 40 g·kg^−1^. Mice in the SE group were sacrificed by cervical dislocation after 4, 8, 12, 16, 24, and 36 h following the administration of gavage, while mice in the SEP group were sacrificed after 2, 4, 8, 12, 24, and 36 h. There are six mice in each group at each time point. The heart, liver, spleen, lung, kidney, and colon tissues were quickly dislodged, and the processed tissue samples were stored at −20°C for further analysis.

### 2.10. Pharmacokinetic Parameters Analysis and Statistical Analysis

There were two groups of SE and SEP samples, with 6 samples in each group. The blood concentration of each sample was detected within the specified time, the pharmacokinetic parameters were analyzed using the DAS software package (version 2.0, Shanghai, China) by noncompartmental model, and the maximum concentration (*C*_max_), time to maximum concentration (*T*_max_), half-life of elimination (T_1/2Z_), area under the concentration time curve (AUC), apparent volume of distribution (V_z/F_), clearance (C_Lz/F_), and mean retention time (MRT) were calculated. A paired *t*-test analysis was conducted using the SPSS software (version 20.0, USA) to test whether these variables are different between the two groups. Data are expressed as mean ± SD.

## 3. Results

### 3.1. Method Validation

#### 3.1.1. Selectivity

**Figure 2:**
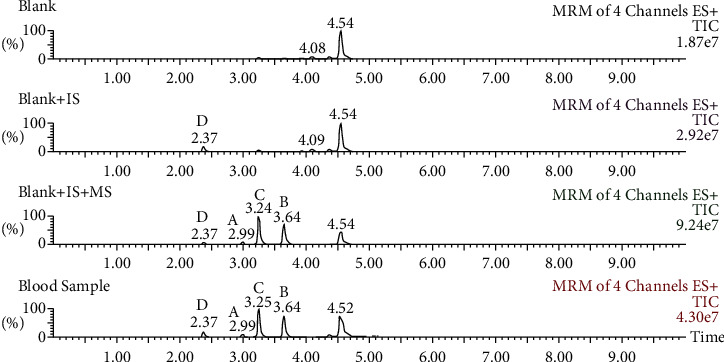
The chromatograms of three components and wogonin. (A, Euphorbia factor L_1_; B, Euphorbia factor L_2_; C, Euphorbia factor L_3_; D, wogonin).

The chromatograms of the three components and wogonin are shown in Figure 2. The chromatographic peaks of Euphorbia factors L_1_, L_2_, and L_3_, and IS were well separated and were not affected by endogenous substances, indicating that the method has good selectivity.

#### 3.1.2. Linearity Range and Limit of Quantitation

Standard curves of the three analytes are summarized in Tables 1 and 2. For each analytical run, the calibration curves of both plasma and tissue samples showed good linearity (*r* >0.99) in the range of 1.002–808.0 ng·mL^−1^. The LOQ of Euphorbia factors L_1,_ L_2_, and L_3_ were 1.002, 1.010, and 1.002 ng·mL^−1^, respectively. These results can provide sufficient sensitivity for subsequent pharmacokinetic studies.

#### 3.1.3. Precision and Accuracy

According to Tables 3 and 4, the RSD of intra- and interday precision were all smaller than 15.0%, while the corresponding accuracy was within 5%. These data have shown that the precision and accuracy of this method are acceptable.

#### 3.1.4. Stability

The stability of the three components in rat plasma and mouse tissues under various conditions is shown in Tables 5 and 6. The RSD values of the three components ranged from 0.15 to 7.81%, indicating that the components in rat plasma and mouse tissues were stable after storage at room temperature for 30 min, at 4°C for 24 h, and three freeze-thaw cycles.

#### 3.1.5. Matrix Effect and Extraction Recovery

The matrix effects and extraction recoveries of the three components in rat plasma and mouse tissues are shown in Tables 7 and 8. All analytes had satisfactory extraction recovery from 95.10–114.14%. Meanwhile, the matrix effect values of the three active ingredients ranged from 84.72–110.35%. These results showed that the values of matrix effect and extraction recovery were within the acceptable range.

#### 3.1.6. Pharmacokinetic Study

**Figure 3:**
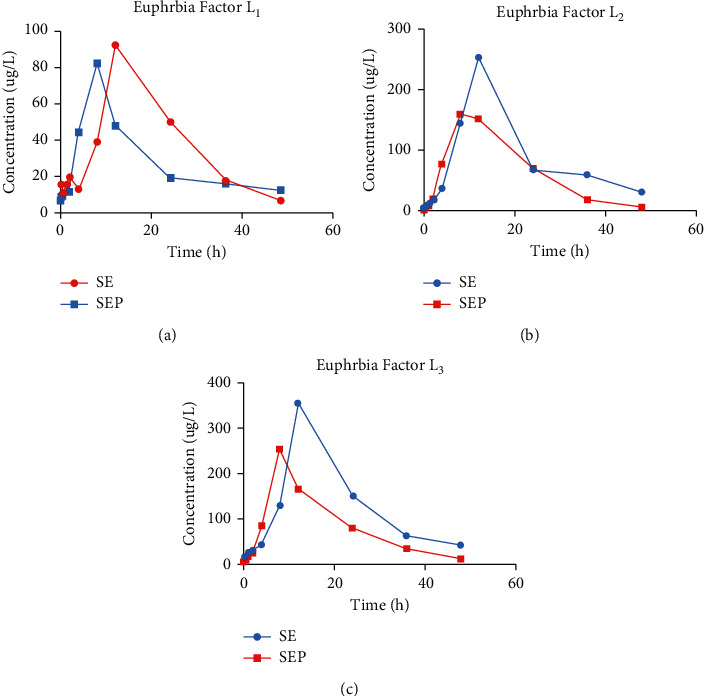
The concentration time curve of Euphorbia factor L_1_ (a), Euphorbia factor L_2_ (b), and Euphorbia factor L_3_ (c) in rat plasma after oral administration of SE or SEP extract (*n* = 6).

The established UPLC-MS/MS method was applied to the pharmacokinetic study of Euphorbia factors L_1_, L_2_, and L_3_ in rat plasma after oral administration of SE and SEP extracts, respectively. The concentration time curves after oral administration of the SE or SEP extracts are shown in Figure 3. The main pharmacokinetic parameters are shown in Table 9.

It can be seen from the chart that the pharmacokinetics of Euphorbia factors L_1_, L_2_, and L_3_ are similar, showing a slow absorption rate after administration. In the crude SE, the *T*_max_ values of three components were more than 10 h. After the administration of SEP extract to rats by gavage, the time to maximum concentration (*T*_max_) of Euphorbia factor L_1_ was significantly less than that for crude SE (*p* < 0.01), and the time to maximum concentration (*T*_max_) of Euphorbia factors L_2_ and L_3_ was also less than that for crude SE (*p* < 0.05), with each *T*_max_ <10 h, indicating that these diterpene esters can reach the maximum blood concentration within 10 h after processing, and they were absorbed more rapidly in *vivo* after processing. After giving the same dose of SE and SEP, the area under the concentration time curve (AUC) of Euphorbia factors L_2_ and L_3_ in SEP group was significantly lower than those of SE group (*p* < 0.05), among which the AUC of Euphorbia factor L_3_ was found to be the highest among the three analytes. The mean retention time (MRT) of Euphorbia factor L_2_ in SEP was lower than that of SE (*p* < 0.05), indicating that the action time shortened in *vivo* after processing. Compared with SE group, the maximum concentration (*C*_max_) of the three components in the blood of SEP group decreased. In terms of the half-life of elimination (T_1/2Z_), the T_1/2Z_ values of the three compounds were 9.53, 9.57, and 9.35 h for SE, but 8.42, 7.42, and 7.46 h for SEP, which suggested that SEP was quickly eliminated while SE was more slowly eliminated.

#### 3.1.7. Tissue Distribution Study

**Figure 4:**
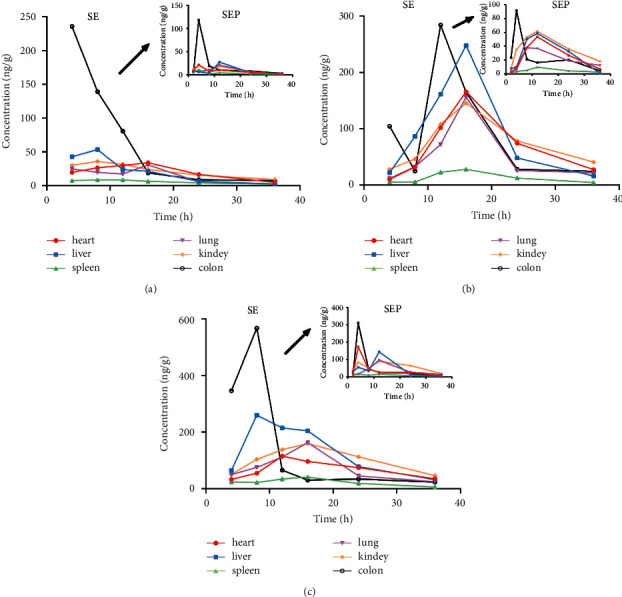
Concentration distribution of Euphorbia factor L_1_ (a), Euphorbia factor L_2_ (b), and Euphorbia factor L_3_ (c) at different time points in tissue samples of mice after oral administration of SE or SEP extract (*n* = 6).

The tissue distribution of Euphorbia factors L_1_, L_2_, and L_3_ after oral administration of the SE or SEP extract was investigated by collecting tissues including the heart, liver, spleen, lung, kidney, and colon tissues. The mean concentration versus time profiles of Euphorbia factors L_1_, L_2_, and L_3_ in the mouse tissues are shown in Figure 4. Based on the experimental results, after mice were administered SE and SEP extracts by gavage, Euphorbia factors L_1_, L_2_, and L_3_ could be detected in the heart, liver, spleen, lung, kidney, and colon tissues. Among which, the highest tissue concentration of Euphorbia factors L_1_, L_2_, and L_3_ in SE group was detected in the colon (235.43 ng·g^−1^, 284.47 ng·g^−1^, 567.52 ng·g^−1^), followed by the liver (53.39 ng·g^−1^, 247.95 ng·g^−1^, 259.69 ng·g^−1^) and the kidney (36.23 ng·g^−1^, 145.52 ng·g^−1^, 159.92 ng·kg^−1^), while the highest tissue concentration of Euphorbia factors L_1_, L_2_, and L_3_ in SEP group was detected in the colon (117.97 ng·g^−1^, 91.44 ng·g^−1^, 310.04 ng·g^−1^), followed by the liver (27.55 ng·g^−1^, 58.37 ng·g^−1^, 143.81 ng·g^−1^) and the kidney (21.55 ng·g^−1^, 61.59 ng·g^−1^, 89.92 ng·kg^−1^). Besides, almost all tissues reached the highest concentration level at the 4 h after administration and then began to decline; the levels of the three analytes in tissues were almost undetectable after 36 h.

## 4. Discussion

### 4.1. Sample Pretreatment Optimization

In the preliminary protein precipitation experiment, the liquid-liquid extraction of ethyl acetate, ether, and n-butanol was studied for sample preparation. With the extraction yield of three characteristic components and the disturbance degree of endogenous substances were used as indices, the results revealed that ethyl acetate had better extraction rate for all analytes in selectivity and sensitivity. Therefore, ethyl acetate extraction method was selected to treat the biological samples.

### 4.2. UPLC-MS/MS Optimization

In our previous study, we determined the retention time, m/z, and ionic strength of chemical composition in SE and SEP using UPLC-MS/MS. The differences in chemical composition were significant between SE and SEP. Using the negative ion mode, the active components detected in SE and SEP were palmitic acid, oleic acid, aesculetin, and euphorbetin, while most lathyrane diterpenoid-type compounds were detected using the positive ion mode [23]. Thus, the positive ion mode was used to analyze the differences between SE and SEP.

### 4.3. Selection of IS

The retention time and extraction recovery of wogonin were similar to that of Euphorbia factors L_1_, L_2_, and L_3_, while the ion response was similar to that of the component to be measured. Wogonin can be completely separated from the three analytes, and the SE and SEP do not contain wogonin. Therefore, wogonin was chosen as an internal standard.

### 4.4. Pharmacokinetic Study

The three active compounds shared some similar pharmacokinetic parameters, which may be due to the similarity of their structures. There are two acetate groups and one phenylacetate group in the structure of diterpene ester, which can be hydrolyzed under the action of plasma esterase. It has been reported that the three ester groups of diterpenes are all hydrolyzed to epoxy lathyrol under the action of esterase [16], and the polarity of the solvent, the strength of the alkali, the concentration of the alkali, the temperature, the time, and other factors all affect the metabolism of the diterpene esters [16, 24]. Therefore, the plasma concentrations of the three diterpene esters decreased rapidly from peak concentrations in a short period of time, which may be caused by the metabolism of the diterpene esters in plasma esterase. In addition, these three components have long T_1/2z_, which may be due to the high binding rate between the drug and serum protein; the lower the concentration of the free drug, the lower the clearance rate of the drug and the longer the half-life time. Besides, all the three analytes have a relatively low *C*_max_, the main reason may be that more than half of the three analytes have been excreted [17], and after 48 hours of intragastric administration of SE and SEP extracts, the three components were almost undetectable in plasma, which is consistent with the research of Fu [16]. However, the results of this experiment are also different from those of Fu and Meng [16, 17]; it is speculated that the different drugs by gavage may be the reason for the difference in experimental results. In this study, petroleum ether extract of SE was given intragastric administration while in the literature study, diterpene ester monomer and ethanol extract of SE were given intragastric administration. The complex components contained in the extract may interact with each other and result in inconsistency with the monomer components in *vivo*, which may be the reason why the literature is inconsistent with our experimental results.

### 4.5. Tissue Distribution Study

The results indicated that Euphorbia factors L_1_, L_2_, and L_3_ had good fat solubility and tissue affinity and underwent a rapid and wide distribution into tissues. However, the time to highest concentrations in various tissues was relatively slow, and the active component concentrations in colon, liver, and kidney tissues were more, followed by that in the heart, lung, and spleen. The total amount of three active compounds in the colon was significantly higher than that in other tissues, demonstrating that SE and SEP were mainly accumulated in the colon and suggesting a potential role of this organ in the metabolism of SE. The concentrations of each compound in different tissues varied greatly, and the concentrations and peak time of the three diterpene esters in the same tissue also differed, which may be related to the difference in blood flow between tissues after drug absorption, the difference in drug composition, the binding force between tissues, and the oral route of drug administration. In addition, the levels of the three analytes in each tissue decreased after processing and the differences were found to be statistically significant at individual time points.

## 5. Conclusion

In our study, a quantitative method for the determination of Euphorbia factors L_1_, L_2_, and L_3_ was established by UPLC-MS/MS. This method was found to be rapid, selective, sensitive, and reliable, with good precision and accuracy, and was applied to determine the pharmacokinetics and assess the distribution characteristics of SE and SEP. Our results revealed the processing could influence the pharmacokinetics and tissue distribution of Euphorbia factors L_1_, L_2_, and L_3_ after oral administration of crude and processed SE. The results from our study could lay a foundation for the clinical use of SE and for further study on the processing mechanism of SE.

## Figures and Tables

**Table 1:** Linear relationship of the three analytes in rat plasma samples.

Analytes	Standard curve	*r*	Linear range (ng·mL^−1^)	LOQ (ng·mL^−1^)
Euphorbia factor L_1_	*Y* = 0.0205841*X* + 0.0190221	0.9990	1.002–801.6	1.002
Euphorbia factor L_2_	*Y* = 0.234217*X* + 0.133849	0.9955	1.010–808.0	1.010
Euphorbia factor L_3_	*Y* = 0.192309*X* + 0.188019	0.9977	1.002–801.6	1.002

**Table 2:** Linear relationship of three analytes in tissue samples of mice.

Analytes	Tissue	Standard curve	*r*	Linear range (ng·mL^−1^)	LOQ (ng·mL^−1^)
Euphorbia factor L_1_	Heart	*Y* = 0.034618*X* + 0.00207128	0.9932	1.002–801.6	1.002
	Liver	*Y* = 0.00977173*X* + 0.00916433	0.9990		
	Spleen	*Y* = 0.117309*X* + 0.00187417	0.9980		
	Lung	*Y* = 0.0817354*X* + 0.0872459	0.9976		
	Kidney	*Y* = 0.00450416*X* + 0.000260721	0.9987		
	Colon	*Y* = 0.0349301*X* + 0.022299	0.9976		

Euphorbia factor L_2_	Heart	*Y* = 0.400486*X* + 0.23513	0.9978	1.010–808.0	1.010
	Liver	*Y* = 0.188429*X* + 0.0965572	0.9935		
	Spleen	*Y* = 1.0453X−0.17471	0.9955		
	Lung	*Y* = 0.502219*X* + 0.344347	0.9980		
	Kidney	*Y* = 0.294794*X* + 0.2747	0.9922		
	Colon	*Y* = 0.313598*X* + 0.332799	0.9924		

Euphorbia factor L_3_	Heart	*Y* = 0.250723*X* + 0.120749	0.9959	1.002–801.6	1.002
	Liver	*Y* = 0.192895*X* + 0.0424932	0.9983		
	Spleen	*Y* = 0.581293X−0.0697721	0.9985		
	Lung	*Y* = 0.346932*X* + 0.11191	0.9953		
	Kidney	*Y* = 0.207445*X* + 0.0897848	0.9970		
	Colon	*Y* = 0.281536*X* + 0.176594	0.9979		

**Table 3:** Precision and accuracy of the three analytes in rat plasma samples.

Analytes	Concentration (ng·mL^−1^)	RSD (%)		RE (%)
		Intra-assay precision	Interassay precision	Accuracy
Euphorbia factor L_1_	25	1.68	2.98	0.09
	100	5.12	2.68	0.13
	400	1.85	2.40	0.13

Euphorbia factor L_2_	25	1.35	2.99	−0.24
	100	1.40	11.07	−0.84
	400	2.10	6.01	0.23

Euphorbia factor L_3_	25	1.39	2.83	0.12
	100	2.00	2.31	−0.50
	400	1.35	6.39	−1.49

**Table 4:** Precision and accuracy of the three analytes in tissue samples of mice.

Samples	Concentration (ng·mL^−1^)	RSD (%)		RE (%)
		Intra-assay precision	Interassay precision	Accuracy
Euphorbia factor L_1_	10	2.40	2.37	0.48
	200	0.81	1.33	−0.39
	600	0.89	0.91	−0.21

Euphorbia factor L_2_	10	2.63	5.19	0.09
	200	0.84	1.96	0.03
	600	1.18	1.60	0.09

Euphorbia factor L_3_	10	1.98	1.95	−0.07
	200	0.87	1.05	−0.58
	600	1.16	1.27	−0.85

**Table 5:** Stability of the three analytes in rat plasma samples.

Analytes	Concentration (ng·mL^−1^)	RSD (%)		
		At room temperature for 30 min in plasma	At 4°C for 24 h in plasma	After three freeze-thaw cycles in plasma
Euphorbia factor L_1_	25	7.14	2.96	1.01
	100	1.16	2.18	2.69
	400	1.74	1.35	3.08

Euphorbia factor L_2_	25	1.79	5.99	4.68
	100	3.57	0.97	1.93
	400	2.08	1.46	1.83

Euphorbia factor L_3_	25	1.81	3.60	3.97
	100	1.41	0.69	2.12
	400	1.56	2.73	1.86

**Table 6:** Stability of the three analytes in tissue samples of mice.

Analytes	Concentration (ng·mL^−1^)	RSD (%)		
		At room temperature for 30 min in tissue	At 4°C for 24 h in tissue	After three freeze-thaw cycles in tissue
Euphorbia factor L_1_	10	3.43	0.81	3.25
	200	1.97	3.06	1.59
	600	0.15	4.33	1.80

Euphorbia factor L_2_	10	2.38	1.13	7.81
	200	2.05	0.99	1.19
	600	0.71	1.40	2.35

Euphorbia factor L_3_	10	1.86	3.56	1.41
	200	3.41	1.99	4.68
	600	0.18	1.94	4.06

**Table 7:** Recovery and matrix effect of the three analytes in rat plasma samples.

Analytes	Concentration (ng·mL^−1^)	Mean (%)		RSD (%)	
		Matrix effect	Extraction recovery	Matrix effect	Extraction recovery
Euphorbia factor L_1_	25	104.42	101.17	3.61	3.81
	100	89.15	99.84	1.26	4.09
	400	95.97	100.79	1.94	2.83

Euphorbia factor L_2_	25	110.35	105.83	1.88	5.45
	100	99.24	101.85	1.80	5.10
	400	109.95	98.89	0.85	1.46

Euphorbia factor L_3_	25	92.64	114.14	1.83	1.33
	100	84.72	111.71	1.45	4.00
	400	94.07	112.72	1.24	0.80

**Table 8:** Recovery and matrix effect of the three analytes in tissue samples of mice.

Analytes	Concentration (ng·mL^−1^)	Mean (%)		RSD (%)	
		Matrix effect	Extraction recovery	Matrix effect	Extraction recovery
Euphorbia factor L_1_	10	101.57	98.50	0.95	1.96
	200	104.00	100.67	0.46	1.14
	600	99.63	102.7	0.76	1.63

Euphorbia factor L_2_	10	98.97	104.07	1.69	0.76
	200	101.10	97.10	1.89	2.27
	600	105.40	95.10	0.59	2.01

Euphorbia factor L_3_	10	99.33	101.00	3.29	2.77
	200	102.40	98.23	2.71	2.14
	600	103.63	102.47	2.09	1.63

**Table 9:** Main pharmacokinetic parameters of the three analytes in the plasma of rats after the oral administration of SE or SEP extract (*n* = 6).

Data	Euphorbia factor L_1_		Euphorbia factor L_2_		Euphorbia factor L_3_	
	SE	SEP	SE	SEP	SE	SEP
AUC (0-t) (*μ*g·h·L^−1^)	1703.73 ± 554.18	1391.45 ± 663.03	4391.97 ± 1596.69	3192.79 ± 1409.02	6381.91 ± 1428.34^*∗*^	4100.05 ± 1741.94^*∗*^
AUC (0-∞) (*μ*g·h·L^−1^)	2883.63 ± 2458.81	1416.66 ± 662.13	5382.56 ± 1370.29^*∗*^	3230.99 ± 1415.46^*∗*^	9463.33 ± 6415.46	4208.31 ± 1769.18
MRT (0-t) (h)	17.80 ± 2.16	16.78 ± 3.56	17.04 ± 4.18	15.71 ± 2.18	18.83 ± 2.33	16.10 ± 2.56
MRT (0-∞) (h)	22.57 ± 3.87	17.79 ± 3.92	21.73 ± 4.31^*∗*^	16.29 ± 2.42^*∗*^	24.47 ± 7.51	17.50 ± 3.27
T_1/2z_ (h)	9.53 ± 6.43	8.42 ± 2.67	9.57 ± 4.32	7.42 ± 1.05	9.35 ± 4.91	8.46 ± 2.19
*T*_max_ (h)	11.33 ± 1.63^*∗∗*^	6.67 ± 2.07^*∗∗*^	11.33 ± 1.63^*∗*^	8.00 ± 2.53^*∗*^	11.33 ± 1.63^*∗*^	8.00 ± 2.53^*∗*^
V_z/F_ (L·kg^−1^)	220467.01 ± 146342.43	345882.41 ± 248756.73	85495.40 ± 51708.11	117516.85 ± 57982.30	61277.19 ± 49305.43^*∗*^	108497.33 ± 83949.46^*∗*^
CL_z/F_ L·(h·kg^−1^)	15457.44 ± 8978.23	27871.15 ± 18174.36	5927.25 ± 1697.04	10922.36 ± 5049.21	3993.88 ± 1591.83	8591.23 ± 4521.52
*C*_max_ (*μ*g·L^−1^)	102.68 ± 34.75	91.39 ± 47.39	281.32 ± 105.93	218.53 ± 111.66	381.33 ± 139.59	327.20 ± 147.33

SE group compared with SEP group, ^∗^*p* < 0.05, ^∗∗^*p* < 0.01.

## Data Availability

The data used to support the findings of this study are included within the article.
